# Pan-cancer multi-omics analysis identifies SYNGR4 as a novel clinical prognostic biomarker and therapeutic target in lung adenocarcinoma

**DOI:** 10.1007/s12672-026-05319-z

**Published:** 2026-05-30

**Authors:** Yanjiao Hou, Bing Fu, Lifang Zhan, Jun Guan, Hui Tian

**Affiliations:** 1 Medical Integration and Practice Center, Cheeloo College of Medicine, Shandong University, Jinan, Shandong China; 2 Department of Clinical Laboratory, Qilu Hospital of Shandong University Dezhou Hospital, Dezhou, Shandong China; 3 Department of Healthcare Quality and Safety, Qilu Hospital of Shandong University Dezhou Hospital, Dezhou, Shandong China; 4 Department of Gynecology, Obstetrics and Gynecology Hospital of Fudan University, Shanghai, China; 5 Department of Thoracic Surgery, Qilu Hospital of Shandong University, Jinan, Shandong China; 6 Department of Thoracic Surgery, The First Affiliated Hospital of Shandong First Medical University (Shandong Qianfoshan Hospital), Jinan, Shandong China

**Keywords:** SYNGR4, Pan-cancer, Multi-omics, Biomarker, Prognostic, LUAD

## Abstract

**Background:**

While the synaptic vesicle protein SYNGR4 has established roles in the pathobiology of motor neuron disease, its comprehensive oncogenic significance across diverse cancers, and its specific value as a clinical biomarker and therapeutic target in lung adenocarcinoma (LUAD), are entirely unexplored.

**Methods:**

Our pan-cancer multi-omics analysis systematically assessed the expression, prognostic value, prognostic nomogram models, and clinical correlates of SYNGR4, investigated its DNA methylation, genetic alterations, and functional pathways (GO/KEGG/GSEA), and evaluated its associations with immune infiltration, immunotherapy response, and drug sensitivity across various cancer types. In addition, we performed qPCR, CCK-8, colony formation, EdU staining, and wound-healing analyses to elucidate the role of SYNGR4 in LUAD proliferation and migration.

**Results:**

Our pan-cancer multi-omics results indicate that SYNGR4 is upregulated in multiple cancers and correlates with prognosis. Its expression was linked to DNA methylation, immune cell infiltration and improved responses to PD-1 blockade (nivolumab) and anti-PD-L1 therapy. SYNGR4 expression also showed positive correlations with sensitivity to MEK inhibitors (PD-0325901, Trametinib) and Docetaxel. Pathway analysis linked SYNGR4 to cell cycle progression and synthesis of DNA. In addition, knockdown of SYNGR4 via siRNA suppressed the proliferative and migratory capacities of LUAD cells in vitro.

**Conclusion:**

This study establishes SYNGR4 as a novel prognostic biomarker in LUAD, thereby positioning it as a potential therapeutic target with clinical utility.

## Introduction

The high mortality rate associated with cancer constitutes a critical global health issue, imposing immense strain on healthcare systems and societies around the world [1]. Notwithstanding progress in clinical management methodologies encompassing operative resection, chemotherapeutic agents, and molecularly targeted treatments, early diagnosis and prognostic evaluation remain limited [2, 3]. Current treatment modalities often fall short in accurately predicting patient outcomes and managing drug resistance [4]. The complexity of cancer biology, coupled with the emergence of treatment resistance, highlights the critical need to develop novel prognostic biomarker discovery and therapeutic target identification aimed at optimizing clinical care strategies and improving prognostic outcomes.

The SYNGR4 gene, which encodes a synaptogyrin protein, is known to contribute to motor neuron disease, its role across cancers and its potential as a clinical biomarker in LUAD remain unclear. Initially identified in amyotrophic lateral sclerosis (ALS), SYNGR4 was found dysregulated in spinal motor neurons during TDP-43-driven disease onset, implicating its role in neurological pathology [5]. Notably, recent evidence extends SYNGR4’s relevance to oncology, demonstrating its overexpression in breast cancer and association with poor prognosis via tumor-associated macrophage M2 polarization [6].

LUAD, which is classified as a specific histologic variant, belongs to the non-small cell lung cancer spectrum [7]. The pathogenesis of LUAD is driven by significant heterogeneity and a complex spectrum of genetic and epigenetic alterations in tumor progression and metastasis. Emerging evidence suggests that synaptic regulatory genes may exert a critical influence on cancer progression [8, 9]. Given the high incidence and mortality of LUAD worldwide, coupled with the lack of effective early diagnostic biomarkers and therapeutic targets, we focused our subsequent experimental validation on this malignancy.

To elucidate the role of SYNGR4, we first conducted a comprehensive pan-cancer multi-omics analysis, assessing its expression, prognostic value, prognostic nomogram models, clinical correlates, DNA methylation, genetic alterations, and enriched pathways. This analysis was extended to evaluate its associations with immune infiltration, immunotherapy response, and drug sensitivity. Subsequently, functional experiments in LUAD cells—utilizing qPCR, CCK-8, colony formation, EdU staining, and wound-healing assays following SYNGR4 knockdown—were performed to confirm its biological impact. Collectively, this study provides the first comprehensive evidence implicating SYNGR4 in LUAD, highlighting its dual utility for both prognosis and therapy.

## Materials and methods

### Acquiring research data

We collected transcriptomic profiles and clinical data encompassing 33 distinct tumor types from TCGA database (https://portal.gdc.cancer.gov/) [10]. For comparison, this tumor data was complemented with normal tissue expression data from the GTEx database (https://www.gtexportal.org/home/) [11]. Differential expression profiling of SYNGR4 was then performed by analyzing these combined TCGA-GTEx datasets. Protein structural characterization, subcellular distribution patterns, and immunohistochemical visualization data were procured from the HPA repository (https://www.proteinatlas.org/).

### Prognostic significance and clinical correlates of SYNGR4 in pan-cancer

We analyzed SYNGR4 expression associations with key clinical correlates in pan-cancer TCGA datasets, including survival outcomes (OS, DSS, PFI), TNM staging, residual tumor status, histological grade, hemoglobin levels, treatment response, and comprehensive pathological staging.

### Prognostic nomogram models

The proportional hazards assumption was examined using the survival package. Univariate and multivariate Cox regression analyses were carried out to identify independent prognostic indicators. Candidate variables were selected based on statistical significance in univariate analysis (*P* < 0.05), clinical accessibility, and absence of significant multicollinearity (variance inflation factor < 3). Based on the multivariate Cox model, prognostic nomograms were established and visualized with the rms package. Stepwise selection based on the Akaike information criterion (AIC) was adopted to determine the final model with optimal fitness. Calibration curves at 1, 3, and 5 years were further generated to evaluate the predictive accuracy and stability of the nomogram models.

### Analysis of genetic alterations, DNA methylation, and drug sensitivity

Genetic alteration analysis were used cBioPortal data ( https://www.cbioportal.org/). DNA methylation analysis were conducted through ULALCAN database (http://ualcan.path.uab.edu/, ) and MethSurv database (https://biit.cs.ut.ee/methsurv/). Pharmacogenomic associations were systematically interrogated leveraging GDSC databases (https://www.cancerrxgene.org/) and CTRP repositories (http://portals.broadinstitute.org/ctrp/) to delineate drug sensitivity associated with SYNGR4 expression.

### Analysis of tumor immune microenvironment and therapy response

We employed the CIBERSORT, ssGSEA, and ESTIMATE algorithms to quantify immune cell subsets in relation to SYNGR4 expression. Correlation analysis between SYNGR4 levels and immunotherapy (anti-PD-1/PD-L1) outcomes was performed using the Kaplan-Meier plotter (https://kmplot.com/analysis/).

### Functional enrichment analysis

Co-expression heatmaps of top 30 up/downregulated LUAD genes correlated with SYNGR4 were generated by R. Stratified GO/KEGG pathway annotation of the top 200 SYNGR4-coexpressed genes (balanced cohort: 100 positively/negatively correlated) delineated cancer-relevant biological processes modulated by SYNGR4. A STRING-based interactome was generated for the hundred most upregulated SYNGR4-coexpressed genes was constructed, with hub genes identified via Cytoscape (MCODE/CytoHubba). The R software environment was employed to perform Gene Set Enrichment Analysis (GSEA) using transcriptomic data of ACC, BRCA, and LUAD.

### Gene expression analysis by qPCR

RNA isolation was performed with TRIzol (invitrogen, USA). SYBR Green-based assays were used for quantitative PCR (qPCR) on an ABI 7500 platform. Reactions utilized a commercially available master mix (ABclonal, China). The qPCR assays employed the following primer sets: SYNGR4, forward 5’-ACCGACGGCTACCAGAACA-3’ and reverse 5’-GAGCTGGAAGGCTGTCTTGA-3’; GAPDH: forward 5’-GGAGCGAGATCCCTCCAAAAT-3’ and reverse 5’-GGCTGTTGTCATACTTCTCATGG-3’. SYNGR4 knockdown (siRNA: 5’-CUAUGAUGCCUGACAACUATT-3’) was validated by qPCR.

### CCK-8 assay

A549 cells with SYNGR4 knockdown and their corresponding control cells were plated in 96-well plates at a density of 5,000 cells per well. Following 24, 48, and 72 h of culture, CCK-8 reagent (10 µL) was added to the cells, which were then incubated for another 4 h at 37 °C, followed by the measurement of absorbance at 450 nm with a microplate reader (Autobio Diagnostics CO., Ltd., China).

### Analysis of colony formation capacity

Colony formation was assessed by low-density seeding of cells in 6-well plates. After 14 days of culture, the developed colonies underwent subsequent fixation, crystal violet staining, and enumeration for clonogenic potential assessment.

### EdU stainning

A 2-hour pulse of 10 µM EdU was used to treat cells, which were then fixed, permeabilized, and fluorescently labeled via click chemistry. Nuclei are counterstained with Hoechst, and EdU-positive cells are quantified by fluorescence microscopy to measure cell proliferation.

### Cell migration assay

Transfected A549 cells (si-SYNGR4/si-NC) maintained under standard DMEM/10% FBS conditions were subjected to a wound-healing assay. Following 24 h culture in 6-well plates, 100 µl pipette-tip wounds were generated and monitored at 0/24/48 h using inverted microscope.

### Statistical analysis

We conducted the analyses using the referenced databases and R software (v4.3.3; RStudio 2024.04.2). GraphPad Prism 8.0 (GraphPad Software, San Diego, CA, USA) was used for data visualization and statistical analysis of all in vitro experimental results. Comparisons between two groups (si-NC vs. si-SYNGR4) were performed using unpaired two-tailed Student’s t-test. Comparisons among multiple groups (BEAS-2B vs. A549 vs. NCI-H1299) were analyzed using one-way analysis of variance (ANOVA) followed by Tukey’s post-hoc test. In this study, asterisks (*, **, ***) corresponded to *P* values of < 0.05, < 0.01, and < 0.001, respectively, with the increasing number of symbols representing greater significance.

## Results

### SYNGR4 expression in pan-cancer

**Fig. 1:**
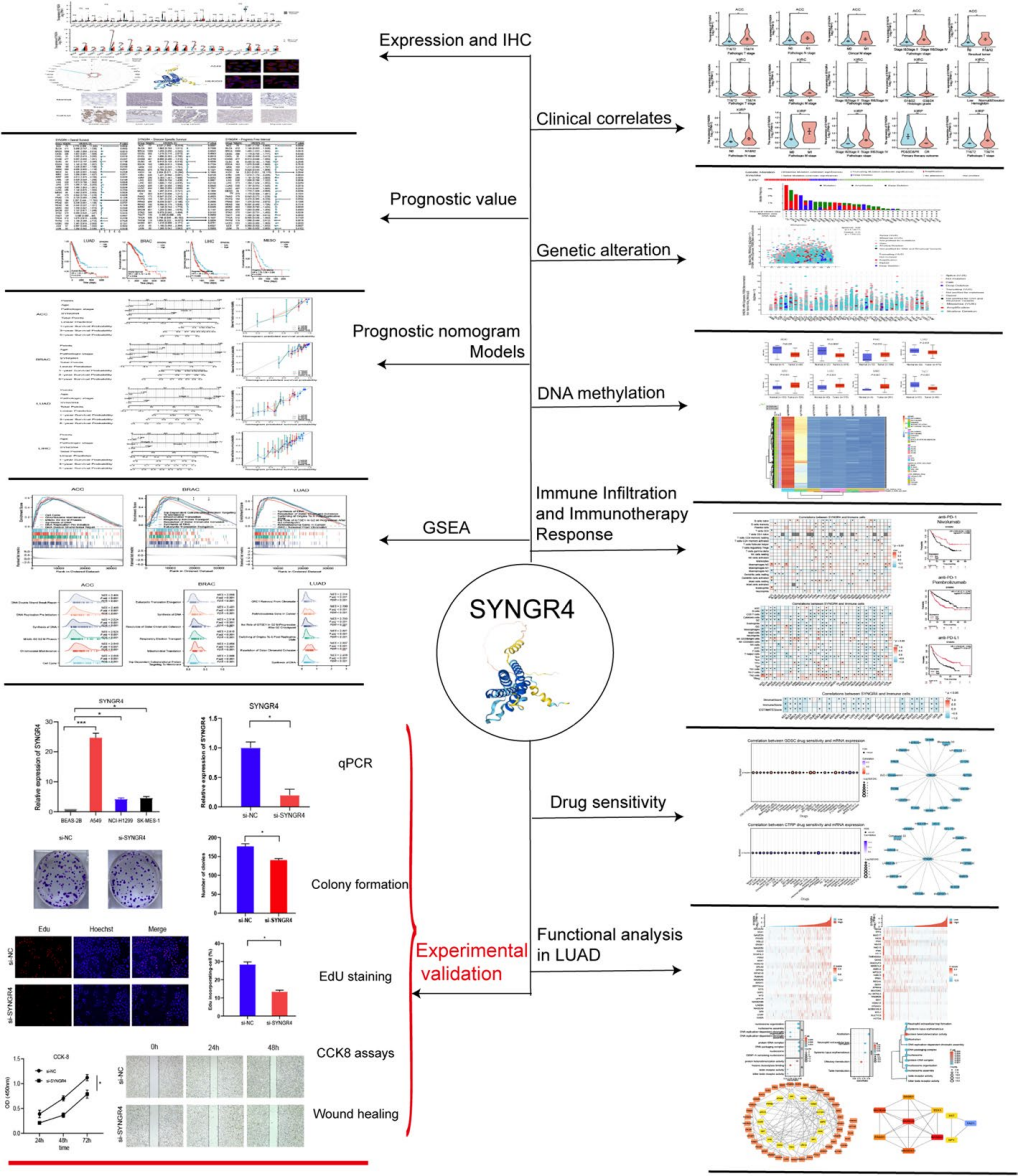
Study flowchart

**Fig. 2:**
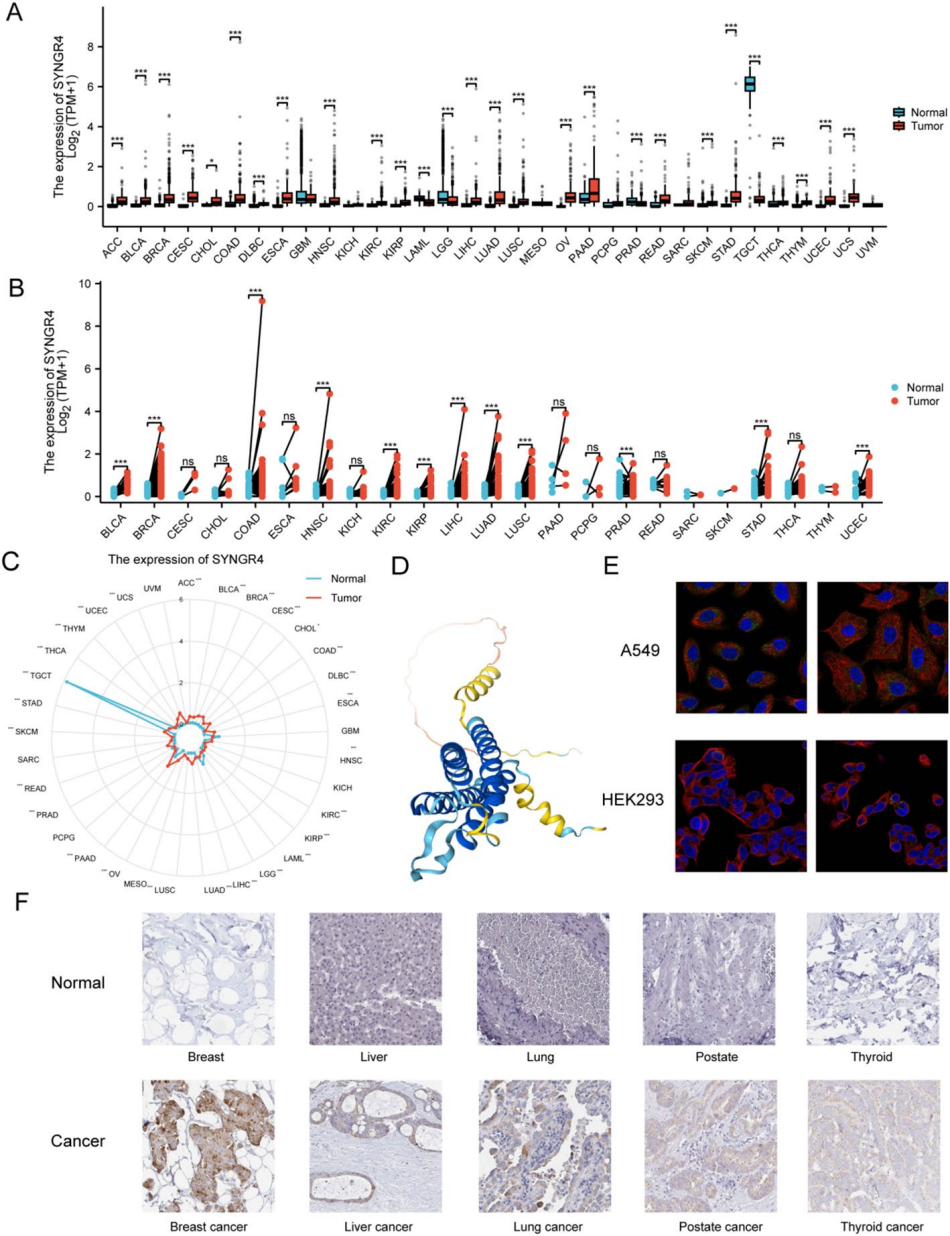
SYNGR4 mRNA expression landscape in pan-cancer. **A** SYNGR4 mRNA expression levels were assessed across 33 tumor types via integrated TCGA/GTEx genomic datasets. **B** Pan-cancer profiling of SYNGR4 expression in 23 malignancies using paired tumor-normal samples in the TCGA database. **C** Radar charts depicting the comparative expression of SYNGR4 in tumors versus normal tissues across 33 malignancies, with integrated TCGA/GTEx data. **D** Molecular structure of SYNGR4. **E** Subcellular localization of SYNGR4 in A549 and HEK293 cells. **F** SYNGR4 immunohistochemical staining in tumor vs. normal tissues.(*P < 0.05, ***P < 0.001)

As shown in Fig. 1, the study’s flowchart is depicted. Primarily, using integrated data from TCGA-GTEx repositories encompassing 33 distinct tumor cohorts, comprehensive analysis demonstrated differential SYNGR4 expression across malignancies. Specifically, 23 types of cancer tissues exhibited elevated SYNGR4 expression, while three types showed reduced expression; the remaining cancer types displayed no significant difference compared to normal tissues (Fig. 2A). Additionally, SYNGR4 expression was analyzed in 23 tumor types using paired samples from the TCGA database (Fig. 2B). To provide a comprehensive overview, a radar chart was conducted to illustrate the SYNGR4 expression across the 33 tumor types relative to normal tissues (Fig. 2C).

Structural data were obtained from the HPA database (Fig. 2D). Additionally, the subcellular localization of SYNGR4 was examined in A549 and HEK 293 cells (Fig. 2E). Futhermore, comparative IHC profiling was performed on histologically normal and tumorous tissues collected from the HPA database for analysis. Analysis of tumor versus normal pairs revealed a consistent transcriptional upregulation of SYNGR4 across several malignancies, notably breast, liver, lung, prostate, and thyroid cancers (Fig. 2F).

### Prognostic value and clinical correlates of SYNGR4 in pan-cancer

**Fig. 3:**
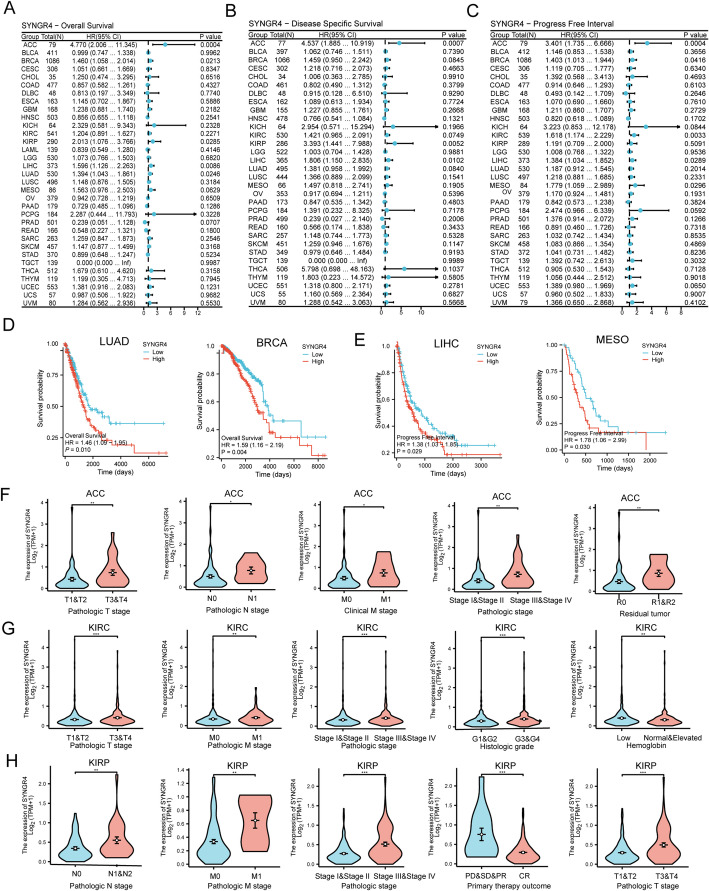
Prognostic significance and clinical correlates of SYNGR4 in pan-cancer. **A–C** Forest plots showing SYNGR4 expression’s impact on OS, DSS, and PFI across cancers. **D**,** E** Kaplan-Meier analyses revealed significant differences in OS (LUAD, BRCA) and PFI (LIHC, MESO) between high/low SYNGR4 groups. **F–H** SYNGR4 correlated with advanced pathological stages (T/N/M) and clinical outcomes in ACC (tumor stage), KIRC (histologic grade), and KIRP (therapy response)

Significant associations of SYNGR4 with OS, DSS, and PFI (Fig. 3A–C) in pan-cancer demonstrate its prognostic value. Forest plot analysis (Fig. 3A) showed that high SYNGR4 expression were significantly correlated with poorer OS outcomes in adrenocortical carcinoma (ACC), breast cancer (BRCA), liver cancer (LIHC), and LUAD (*P* < 0.05, HR > 1). Additionally, SYNGR4 was associated with poor DSS in ACC, kidney renal papillary cell carcinoma (KIRP), and LIHC (*P* < 0.05, HR > 1) (Fig. 3B). SYNGR4 also emerged as as an adverse prognostic indicator for PFI (Fig. 3C) in BRCA, ACC, kidney renal clear cell carcinoma (KIRC), LIHC, KIRP, and mesothelioma (MESO) (*P* < 0.05, HR > 1).

The investigation showed a close association between SYNGR4 gene expression levels and prognosis in seven tumor types within the publicly available TCGA repository, including ACC, BRCA, LIHC, LUAD, KIRC, KIRP, and MESO. SYNGR4 expression was significantly associated with overall survival (OS) in LUAD and BRCA, and with progression-free interval (PFI) in LIHC and MESO; these four representative cancer types were therefore selected for Kaplan-Meier plotting (Figs. 3D, E). Furthermore, SYNGR4 showed prominent clinicopathological correlations in multiple cancers. For concise illustration, three representative cancer types (ACC, KIRC, KIRP) with the most significant associations are shown in Figs. 3F–H. In ACC, this molecular biomarker exhibited significant associations with tumor progression indicators including TNM classification parameters (pT-stage, pN-stage), clinical N stage, pathological stage, and pathological tumor status (Fig. 3F). The expression of SYNGR4 in KIRC was correlated with TNM classification (T and M stages), pathologic stage, hemoglobin and histologic grade (Fig. 3G). SYNGR4 expression levels in KIRP demonstrated significant associations with clinicopathological parameters including Pathological stage, TNM classification (T, N and M stages), and Primary therapy outcome (Fig. 3H).

### Development and assessment of prognostic nomogram models

**Fig. 4:**
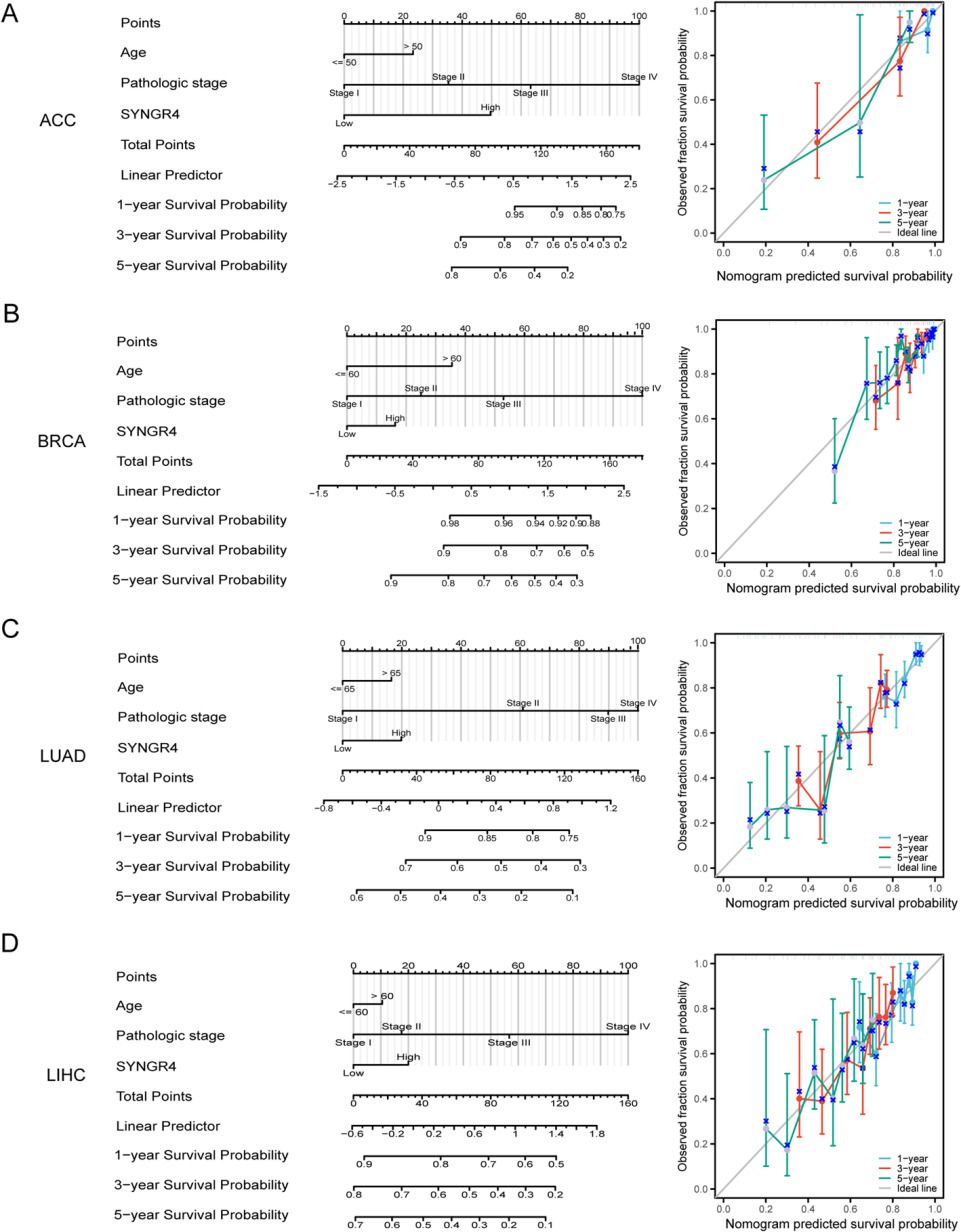
Development and validation of prognostic nomogram models in ACC, BRCA, LUAD and LIHC. **A–D** Construction of predictive models integrating SYNGR4 expression with calibration curves at 1, 3, and 5 years in ACC, BRCA, LUAD and LIHC

The prognostic value of SYNGR4 in specific malignancies was assessed by univariate Cox regression analysis of overall survival. Based on these results, we constructed prognostic nomogram models incorporating age, pathologic stage, and SYNGR4 expression as prognostic factors. These nomograms effectively predicted 1-, 3-, and 5-year survival probabilities and demonstrated significant clinical utility in adrenocortical carcinoma (ACC) (Fig. 4A), breast invasive carcinoma (BRCA) (Fig. 4B), LUAD (Fig. 4C), and liver hepatocellular carcinoma (LIHC) (Fig. 4D).

### Genetic alteration analysis

**Fig. 5:**
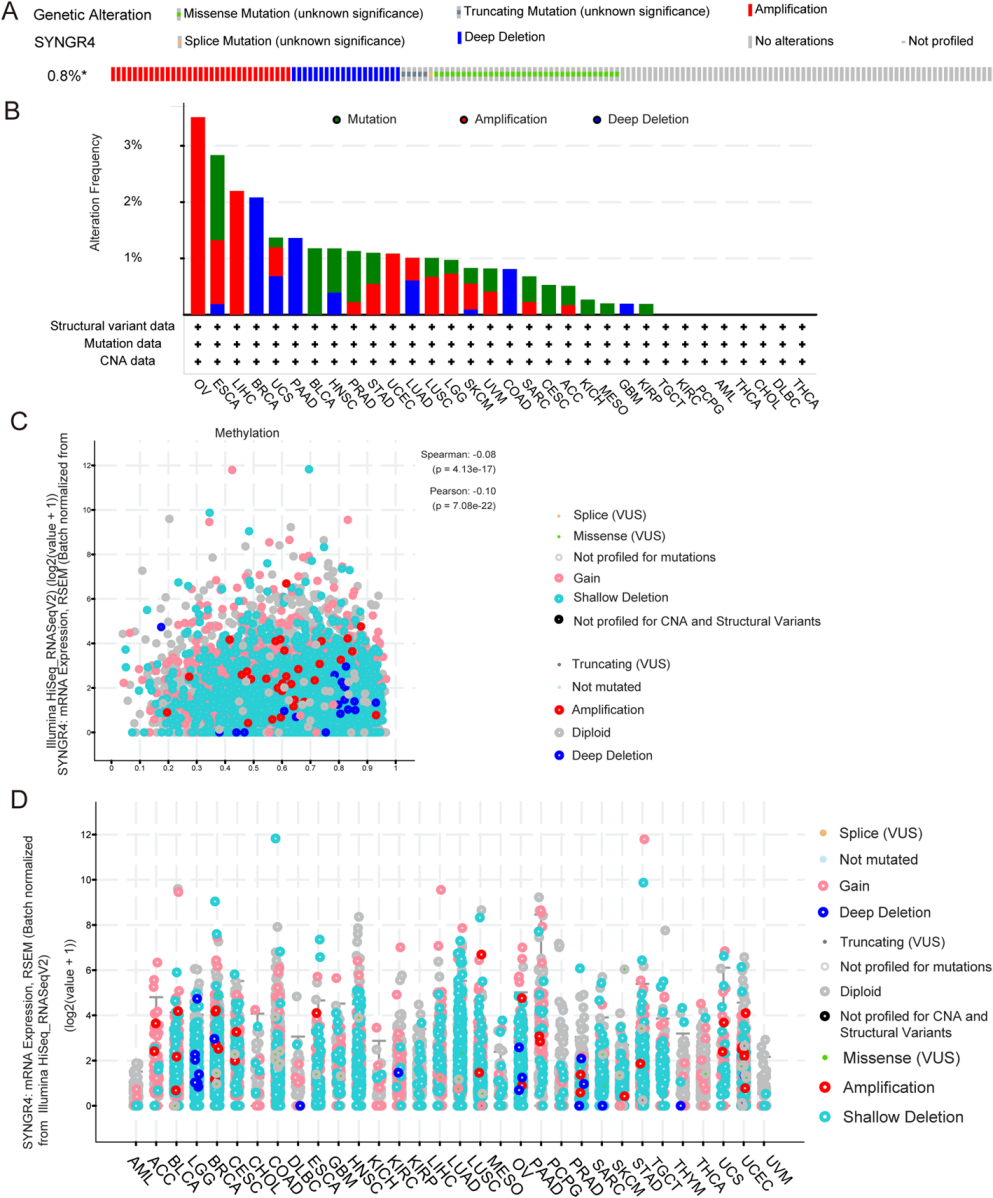
Genetic alteration analysis of SYNGR4 across human cancers. **A** Prevalence of SYNGR4 alterations in pan-cancer samples, with an overall alteration frequency of 0.8%. **B** Distribution of SYNGR4 mutation types and frequencies among different cancer types. **C** Correlation between SYNGR4 methylation status and its mRNA expression levels in various malignancies. **D** Association between putative copy-number alterations (CNA) of SYNGR4 and its transcript abundance in pan-cancer tissues

To profile the genetic landscape of SYNGR4 in human cancers, we queried the cBioPortal database. Analysis revealed alterations in SYNGR4 across multiple tumor types (Fig. 5A), occurring in approximately 0.8% of all profiled samples. Among 32 cancer types examined (Fig. 5B), SYNGR4 exhibited recurrent mutations and deep deletions—the most frequent copy number variation (CNV) events—particularly LUAD.

We further evaluated epigenetic and genomic influences on SYNGR4 expression. In Fig. 5C, the scatter plot shows a clear downward trend: SYNGR4 mRNA expression decreases as DNA methylation level increases, supporting the significant inverse correlation (Spearman *r* = − 0.08, *P* = 4.13e-17; Pearson *r* = − 0.10, *P* = 7.08e-22). Notably, samples with amplification (red points) cluster at higher expression levels, while those with deep deletions (blue points) fall at the lower end of the expression range. In Fig. 5D, stratified plots demonstrate that tumors with copy number gain or amplification generally exhibit higher SYNGR4 expression, whereas those with deep deletions show lower expression. Together, these visual patterns indicate that SYNGR4 expression in tumors is correlated with both genetic and epigenetic alterations.

### DNA methylation of SYNGR4

**Fig. 6:**
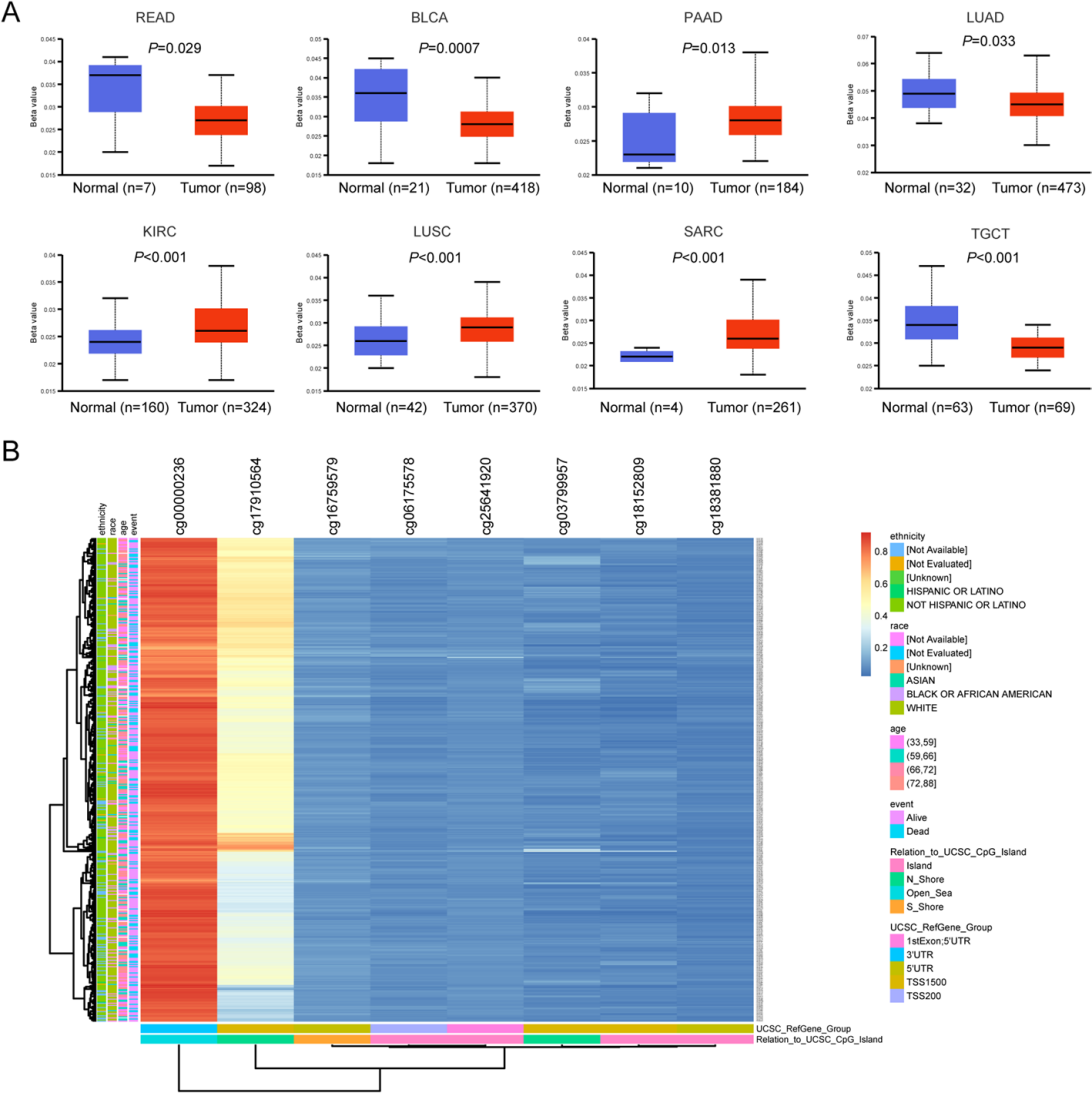
DNA methylation landscape of SYNGR4. **A** Differential promoter methylation (tumor vs. normal) in a pan-cancer setting (UALCAN). **B** Heatmap visualization of CpG site-specific methylation patterns of SYNGR4 in LUAD (MethSurv)

Across a spectrum of cancer types, we profiled SYNGR4 promoter methylation in tumors versus matched normal tissues. Analysis revealed cancer-specific hypermethylation in PAAD, KIRC, LUSC, and SARC, whereas hypomethylation was observed in READ, BLAC, LUAD, and TGCT (Fig. 6A). Further examination using the MethSurv database identified eight specific CpG sites within the SYNGR4 gene that display differential methylation patterns in LUAD (Fig. 6B).

### SYNGR4 modulates immune microenvironment diversity and predicts PD-1/PD-L1 blockade responses in pan-cancer

**Fig. 7:**
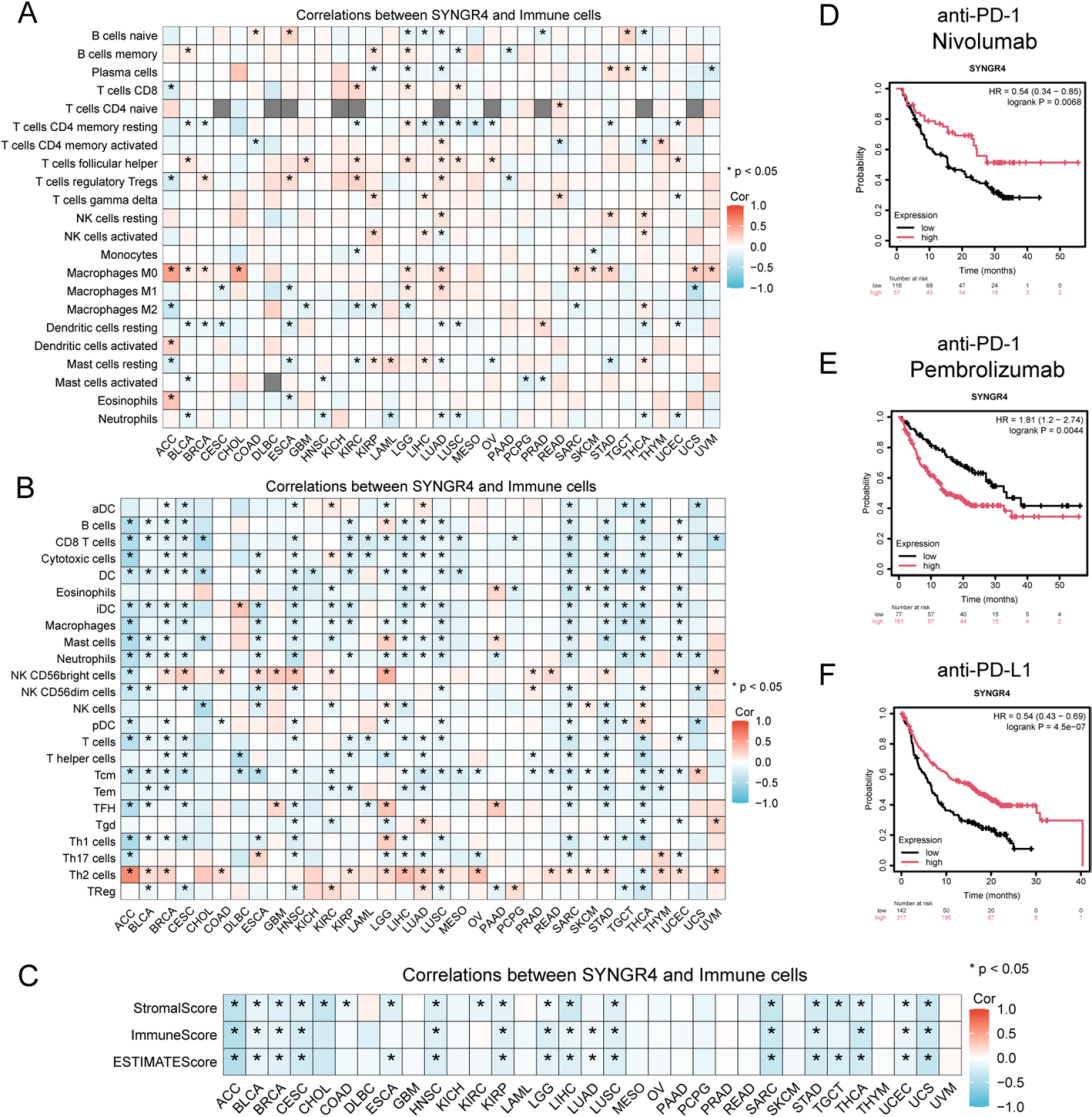
SYNGR4 modulates immune microenvironment diversity and predicts PD-1/PD-L1 blockade responses in pan-cancer. **A** Immune infiltration relationships with SYNGR4 expression quantified by CIBERSORT in multiple cancers. **B** Immune infiltration relationships with SYNGR4 expression quantified by ssGSEA in multiple cancers. **C** Heatmap depicting SYNGR4-immune infiltration correlations using composite ESTIMATE metrics. **D–F **SYNGR4 association with efficacy of anti-PD-1/PD-L1 blockade: **D** nivolumab; **E** pembrolizumab; **F** anti-PD-L1

SYNGR4 expression exhibited cancer-type-specific immune associations through CIBERSORT/ssGSEA/ESTIMATE analyses. Key correlations included M0 macrophages in ACC/CHOL/UCS/UVM, resting mast cells in LAML, and activated NK cells in KIRP (Fig. 7A). Th2 cell associations emerged in ACC/LIHC/OV, while LGG showed concurrent links with NK CD56 bright cells, TFH, and Th1/Th2 cells (Fig. 7B). ESTIMATE analysis revealed significant Stromal-Immune score inverse correlations in 14 malignancies including BRCA, HNSC, and STAD (Fig. 7C).

Immunotherapy response analysis demonstrated SYNGR4’s predictive value: high expression correlated with favorable nivolumab (anti-PD-1) outcomes but inferior pembrolizumab response (Fig. 7D-E). Notably, SYNGR4-high patients showed enhanced anti-PD-L1 therapeutic efficacy (HR = 0.54, *P* = 4.5e-07; Fig. 7F).

### SYNGR4 unveils cancer-specific pathway activation and predictive drug sensitivity

**Fig. 8:**
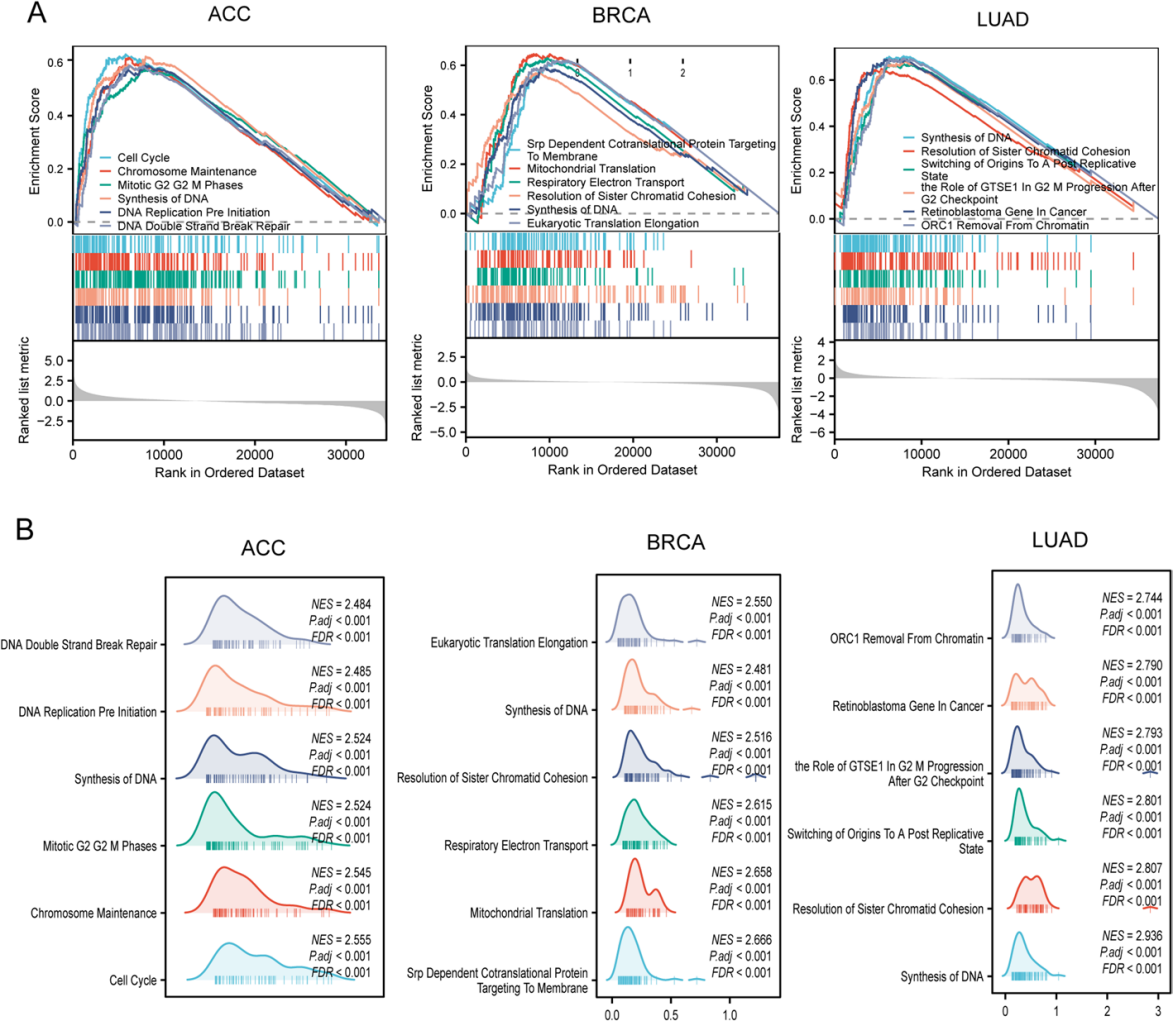
GSEA revealed significant enrichment pathways. **A, B** GSEA was performed based on differential SYNGR4 expression in ACC, BRCA, and LUAD, respectively

**Fig. 9:**
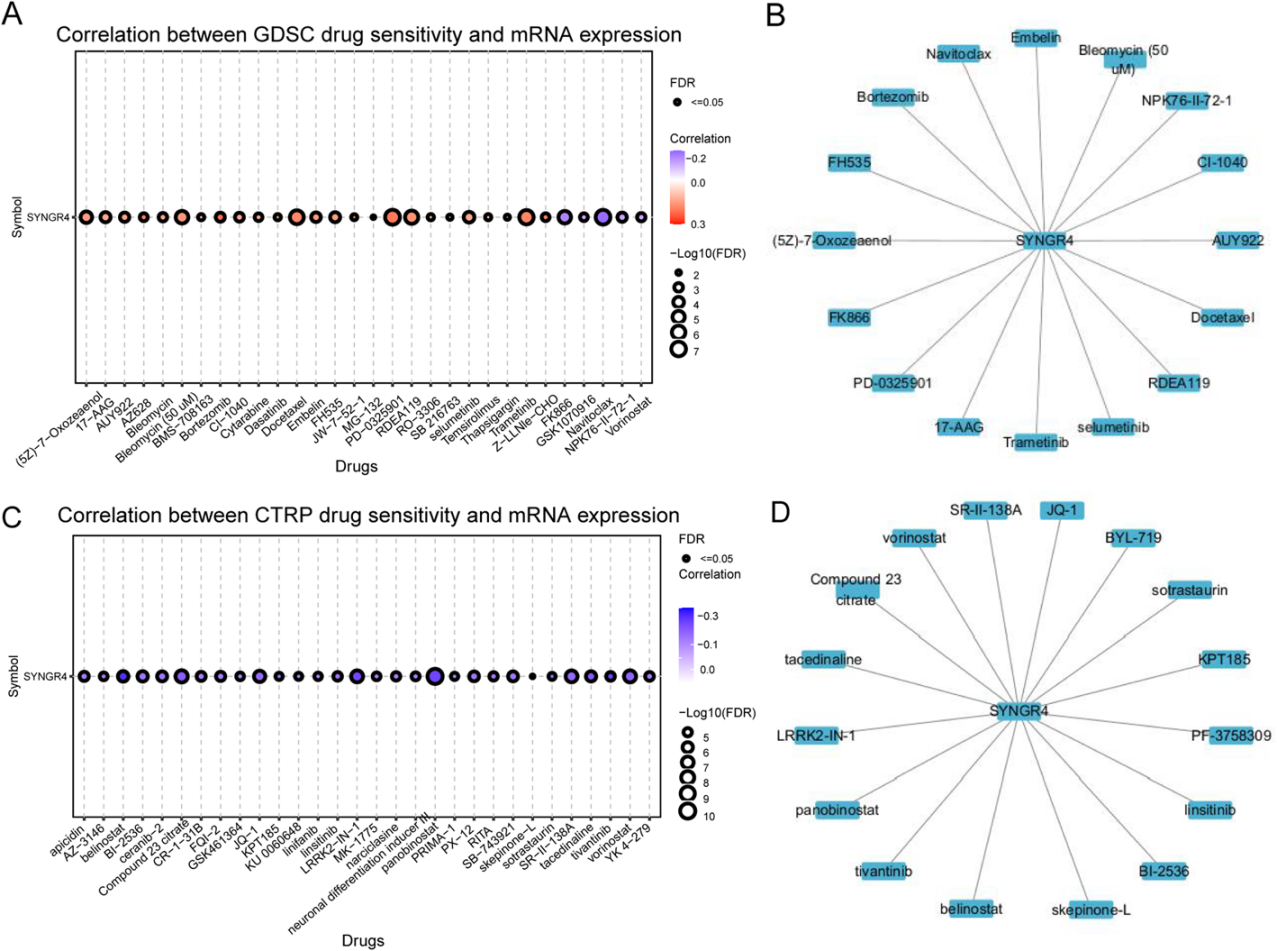
Drug sensitivity analysis. **A** Pan-cancer correlation of GDSC drug response with SYNGR4 mRNA levels. **B** FDA-approved anti-cancer drugs targeting SYNGR4 via GDSC screening. **C** CTRP-based chemotherapy response linked to SYNGR4 expression. **D** FDA-listed drugs with SYNGR4 sensitivity in CTRP analysis

GSEA in prognostically significant cancers (ACC/BRCA/LUAD) revealed SYNGR4-associated pathway enrichments: cell cycle and synthesis of DNA (Fig. 8A, B). Pan-cancer drug sensitivity analysis identified SYNGR4-positive correlations with MEK inhibitors (PD-0325901, Trametinib) and Docetaxel, versus negative associations with apoptosis inducers (Navitoclax, FK866) and HDAC inhibitors (panobinostat, Vorinostat) across GDSC/CTPR datasets (Fig. 9A-D). These findings position SYNGR4 as both a mechanistic regulator of oncogenic pathways and a predictive biomarker for therapeutic response stratification.

### SYNGR4 co-expression networks and functional profiling in LUAD

**Fig. 10:**
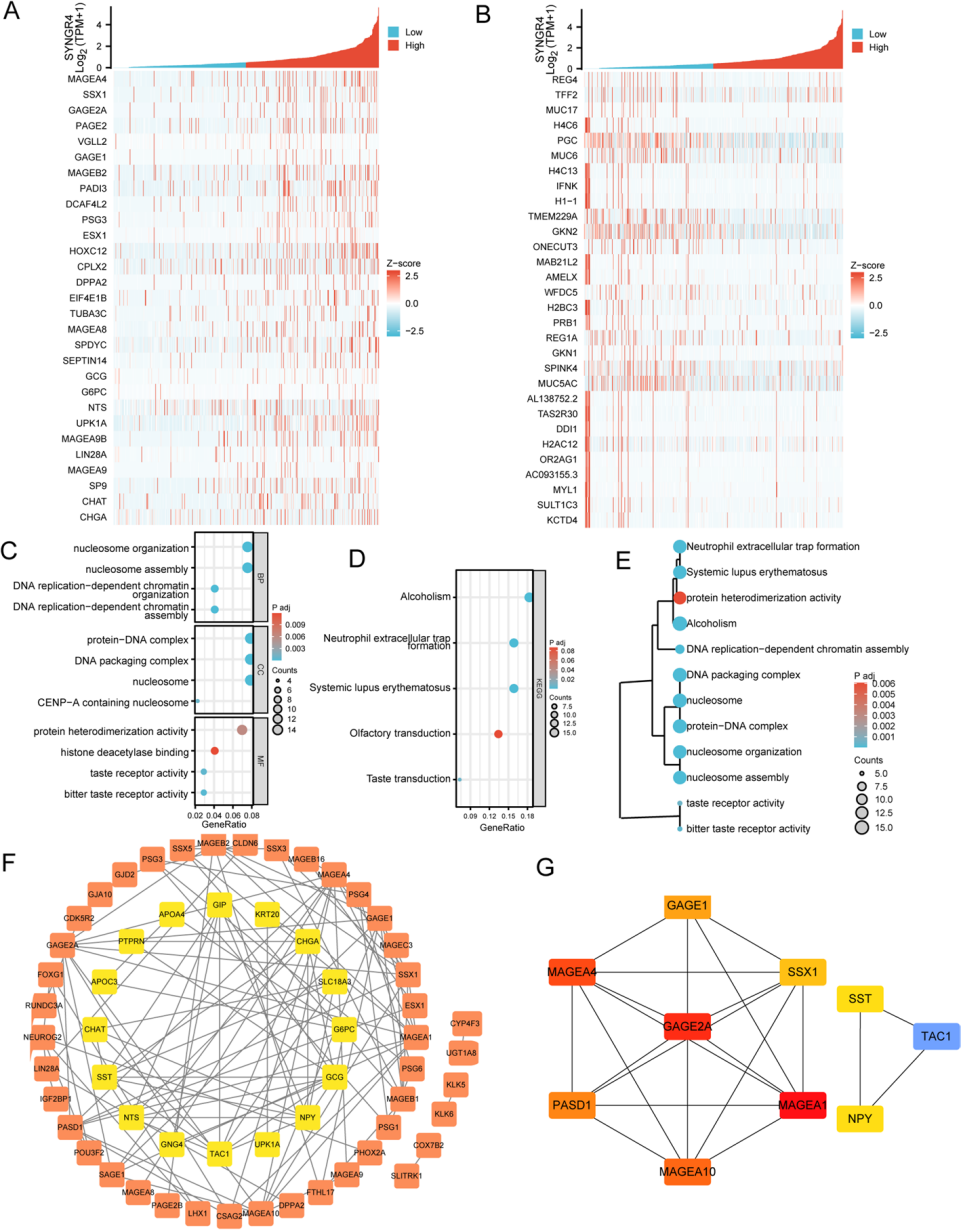
SYNGR4 co-expression networks and functional profiling in LUAD. **A**,** B** Expression patterns of the 30 most significantly co-expressed genes displaying positive (**A**) and negative (**B**) associations with SYNGR4 in LUAD. **C–E** GO and KEGG analyses of SYNGR4 alongside its top 200 co-expressed genes. **F** STRING-derived PPI network comprising the top 100 genes positively co-expressed with SYNGR4. **G** Top 10 hub genes identified through network topology analysis of the PPI interactome

Among these, LUAD attracted our attention due to its significant upregulation of SYNGR4 (Fig. 2A, B), its strong association with poor prognosis (Fig. 3A–C), and the high clinical relevance of identifying novel biomarkers for this common and aggressive malignancy. Analysis of TCGA data identified SYNGR4-coexpressed genes in LUAD, with heatmaps (Fig. 10A, B) highlighting the top 30 positively/negatively correlated genes. Gene ontology and pathway enrichment analyses were conducted among the top 200 co-expressed genes (GO/KEGG; Fig. 10C–E) revealed SYNGR4’s association with nucleosome organization, chromatin assembly, and DNA packaging. PPI network analysis of SYNGR4-correlated genes (Fig. 10F) using STRING identified 10 hub genes—including MAGEA4, PASD1, GAGE1, GAGE2A, MAGEA10, SSX1, MAGEA1, SST, NPY, and TAC1 (Fig. 10G)—as potential key mediators of SYNGR4-regulated biological processes. These findings collectively delineate SYNGR4’s involvement in chromatin remodeling and transcriptional regulation pathways in LUAD.

### Knockdown of SYNGR4 suppresses LUAD cell proliferation and migration

**Fig. 11:**
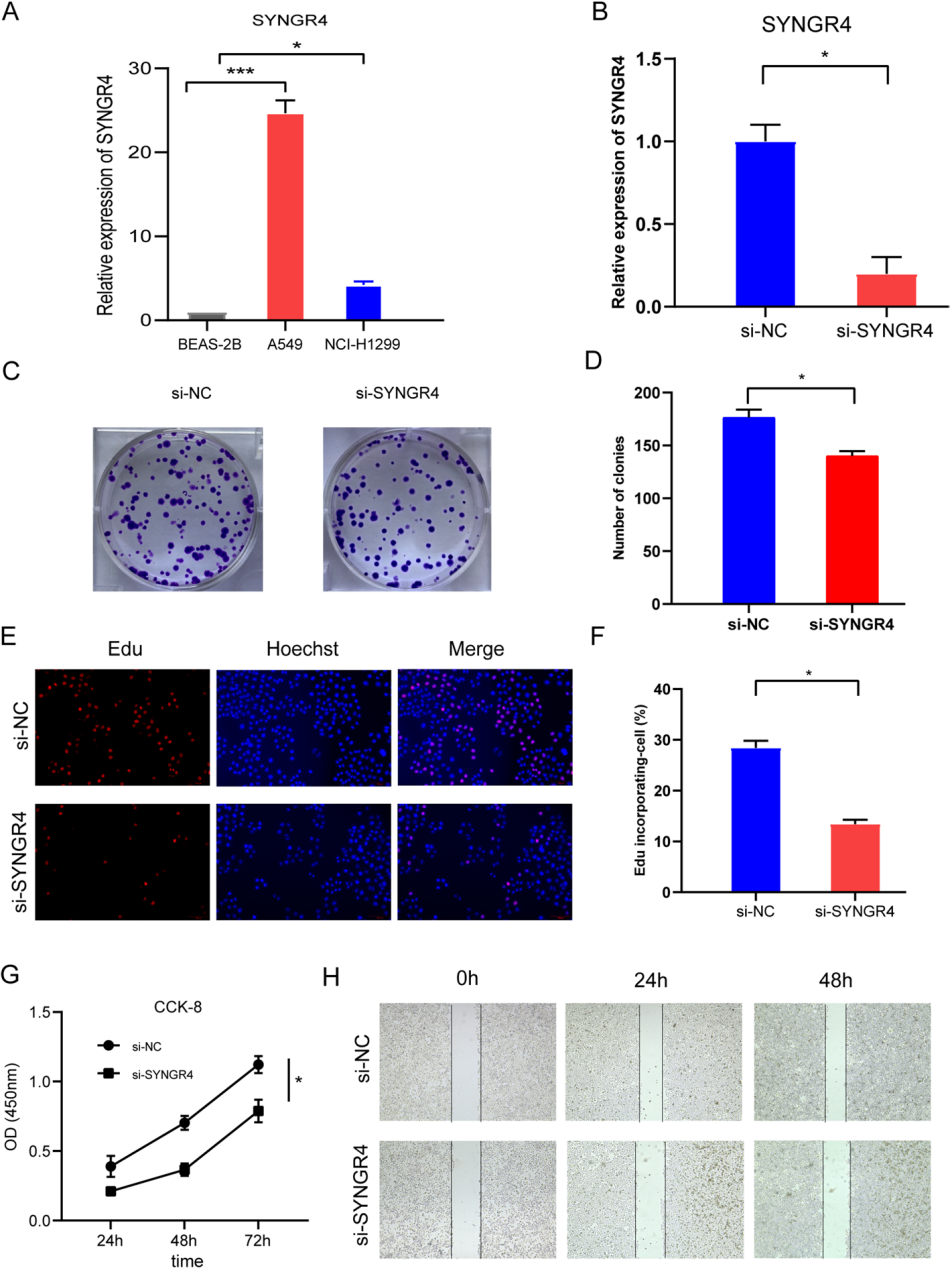
Inhibitory effect of SYNGR4 knockdown on LUAD proliferation and migration. **A** Elevated SYNGR4 mRNA in LUAD cells (A549/NCI-H1299 vs. BEAS-2B normal cells. **B** qPCR validation of SYNGR4 knockdown (KD) efficiency. **C–D** Colony formation assay showing reduced clonogenicity after SYNGR4 knockdown. **E**,** F** EdU staining (red: proliferating cells; blue: nuclei) with quantitative analysis. **G** CCK-8 assay demonstrating impaired proliferation. **H** wound healing assays showing reduced cell migration after SYNGR4 knockdown. (*P < 0.05, ***P < 0.001)

qPCR analysis identified significantly elevated SYNGR4 expression in in LUAD cells (A549/NCI-H1299) relative to BEAS-2B normal cells (Fig. 11A). A549 showed the most significant upregulation of SYNGR4 and was therefore selected for subsequent functional validation experiments. Effective siRNA-mediated SYNGR4 knockdown was confirmed by qPCR (Fig. 11B). Colony formation assays revealed that SYNGR4 knockdown substantially diminished colony numbers compared to controls (Fig. 11C, D). EdU assays further quantified a lower proportion of proliferating cells in SYNGR4-knockdown cells (Fig. 11E, F). Consistent with this, CCK-8 assays demonstrated significantly reduced proliferative capacity in cells with SYNGR4 knockdown across 24-, 48-, and 72-hour time intervals (*P* < 0.05; Fig. 11G). Collectively, these observations highlight SYNGR4 as a pivotal regulator in driving cellular proliferation during LUAD progression. Wound-healing assays revealed impaired migratory capacity in SYNGR4-knockdown cells, with significantly slower scratch closure at 24 and 48 h compared to controls (Fig. 11H), underscoring SYNGR4’s role in promoting the migration of A549 cells.

## Discussion

With the rising incidence of cancer globally, there is a critical imperative to discover effective biomarkers that can enhance diagnosis, prognostication, and treatment strategies [12–17]. Current therapeutic approaches often lack specificity and can lead to suboptimal patient outcomes due to late-stage diagnoses or resistance mechanisms [18–20]. Thus, identifying novel biomarkers that can guide clinical decisions is critical for improving cancer management and patient survival.

Despite its recognized involvement in motor neuron disease, the broader implications of SYNGR4 in oncology—specifically its pan-cancer oncogenic role and its viability as a clinical biomarker or therapeutic target in LUAD—remained an open question, which this study aimed to address. This integrative pan-cancer analysis demonstrates that SYNGR4 holds substantial clinical significance, with validated overexpression in diverse malignancies. Higher SYNGR4 levels predicted worse clinical outcomes, including worse OS, DSS, and PFI in several malignancies, such as ACC, BRCA, and LUAD. Our findings are consistent with the previously reported disease-specific roles of SYNGR4: its oncogenic functions in breast cancer mediated through immune modulation [6]. Importantly, SYNGR4 expression shows significant associations with key clinical correlates in LUAD. The integration of SYNGR4 into clinical nomogram models enhances prognostic accuracy and underscores its potential as a therapeutic target for precision oncology in LUAD.

Collective evidence from pan-cancer proteogenomic analyses indicates that somatic alterations can drive molecular changes in a tumor-type-specific manner, even when their overall frequency is low [21]. This aligns with our observation that SYNGR4, despite a low pan-cancer mutation frequency (0.8%), exhibits prominent mutations and deep deletions in LUAD, suggesting a context-dependent role. Furthermore, the established correlation between copy-number alterations and mRNA levels in such studies supports the notion that genomic instability is a plausible mechanism influencing SYNGR4 transcript abundance.

A well-established observation in oncology is the broad correlation between gene-level protein abundance and corresponding mRNA expression or copy number alterations across tumor types [21]. Our analysis revealed that SYNGR4 exhibits a low mutation frequency (0.8%) across pan-cancer samples, with specific alterations like deep deletions being particularly prominent in LUAD. This low frequency suggests that genetic changes in SYNGR4, while not common, may play a context-specific role in certain malignancies. This observation is consistent with large-scale pan-cancer studies which confirm that while driver alterations in canonical pathways are a universal hallmark of cancer, their extent and molecular mechanisms are highly tumor-type specific [22, 23]. Furthermore, the correlation between copy-number alterations and mRNA expression implies that genomic instability could be one mechanism influencing SYNGR4 transcript levels.

The critical role of epigenetic alterations in cancer is underscored by their ability to rewire gene expression programs under diverse pathophysiological contexts, including hypoxia, metabolic stress, and the maintenance of stemness [24–27]. In line with this broad regulatory potential, our epigenetic analysis further revealed that SYNGR4 promoter methylation exhibits cancer-type-specific patterns, with both hypermethylation and hypomethylation observed across diverse tumors. The identification of specific CpG sites in LUAD highlights the potential regulatory role of DNA methylation in modulating SYNGR4 expression. Collectively, these findings suggest that dysregulation of SYNGR4 in cancer is driven by both genetic and epigenetic mechanisms, underscoring the need for functional studies to elucidate its precise role in tumorigenesis.

Given that TME is shaped by intricate crosstalk between malignant and stromal cells [28–31]and that PD-1 blockade exerts its effect by remodeling this very compartment [32, 33], identifying key regulators of this ecosystem is crucial. Our analysis implicates SYNGR4 as such a regulator, as its pan-cancer expression shows significant correlations with diverse immune cell infiltrates. The context-specific immune associations—with M0 macrophages in ACC and Th2 cells in LIHC—support its potential role in modulating the tumor immune landscape. In LUAD, SYNGR4 expression correlated with infiltration of Treg cells, Th2 cells, and M0/M1 macrophages (Fig. 7A), suggesting a possible role in shaping the immune microenvironment. Pharmacogenomic analysis further linked elevated SYNGR4 to increased sensitivity to MEK inhibitors (PD-0325901, Trametinib) and Docetaxel, and reduced sensitivity to HDAC inhibitors (Fig. 9A-D), offering pharmacogenomic clues for tailored LUAD treatment.

Notably, SYNGR4 expression held distinct predictive value for different immune checkpoint therapies: it predicted enhanced clinical outcomes with nivolumab (anti-PD-1) therapy but reduced efficacy of pembrolizumab. The pronounced therapeutic advantage observed in SYNGR4-high patients receiving anti-PD-L1 regimens further supports its role as a potential biomarker for informing treatment selection with PD-1/PD-L1 inhibitors. This dichotomous effect prompts the hypothesis that SYNGR4 mediates differential regulation of the PD-1/PD-L1 immune checkpoint axis.

As a synaptic vesicle membrane protein, SYNGR4 may regulate intracellular vesicle transport, exosome secretion, and tumor antigen presentation (e.g., MHC-I molecule trafficking), thereby influencing the tumor immune microenvironment of LUAD. In addition, SYNGR4 may affect cytokine release and immune cell infiltration through vesicle-mediated intercellular communication, providing a potential biological basis for its association with immunotherapy efficacy.

Dysregulated cell cycle progression is a hallmark of tumorigenesis [34–38]. The GSEA results revealed significant enrichment of ‘Cell Cycle’ and ‘synthesis of DNA’ pathways in ACC, BRCA, and LUAD, suggesting SYNGR4’s potential role as a key regulator of these processes. The involvement of SYNGR4 in the cell cycle highlights its potential function in facilitating tumor cell proliferation, as dysregulation of cell cycle checkpoints control constitutes a fundamental driver of tumor progression. Additionally, the enrichment of SYNGR4 in DNA synthesis pathways provides direct mechanistic support for its role in promoting LUAD proliferation. The functional link between SYNGR4 and DNA replication provides a plausible explanation for the observed suppression of cell growth upon its knockdown [39–41]. Targeting DNA replication is a established anticancer strategy [42, 43], therefore, SYNGR4 may represent a novel therapeutic vulnerability in LUAD.

Experimental validation confirms SYNGR4’s critical role in LUAD pathogenesis, demonstrating significantly elevated expression in malignant versus non-neoplastic bronchial cells. The consistent impairment in proliferative capacity—evidenced by reduced colony formation, decreased CCK-8 absorbance, and diminished EdU-positive populations upon SYNGR4 knockdown. Notably, the concomitant suppression of migratory capacity in wound-healing assays reveals SYNGR4’s multifunctional role in LUAD progression. These findings demonstrate SYNGR4’s role as an oncogenic driver by enhancing proliferation and migration.

Certain limitations of our work must be acknowledged. First, the retrospective analysis of public omics data may introduce population selection bias due to non-randomized sampling, and the absence of in-depth mechanistic investigations and in vivo animal experiments limits the comprehensive understanding of SYNGR4’s functional roles. Second, clinical validation through prospective cohorts remains crucial to confirm SYNGR4’s therapeutic relevance in oncology. Beyond that, a limitation of our study is that the prognostic nomogram was constructed and internally validated solely on the TCGA cohort. Although ten-fold cross-validation indicated good model robustness, external validation with independent cohorts (e.g., GEO datasets) is still required prior to clinical application. We intend to conduct such validation in future research.

In summary, this integrative pan-cancer multi-omics study, substantiated by experimental validation, establishes SYNGR4 as a novel oncogene and a molecule of clinical promise for prognosis and therapy in LUAD.

## Data Availability

Data supporting the findings of this study are available from the corresponding author upon reasonable request.

## References

[1] Casolino R, Sullivan R, Jobanputra K, Abdel-Wahab M, Grbic M, Hammad N, Kutluk T, Melnitchouk N, Mueller A, Ortiz R, et al. Integrating cancer into crisis: a global vision for action from WHO and partners. Lancet Oncol. 2025;26(1):e55–66. 39708820 10.1016/S1470-2045(24)00522-9

[2] Li X, Jin K, Cheng TC, Liao YC, Lee WJ, Bhullar AS, Chen LC, Rychahou P, Phelps MA, Ho YS, et al. RNA four-way junction (4WJ) for spontaneous cancer-targeting, effective tumor-regression, metastasis suppression, fast renal excretion and undetectable toxicity. Biomaterials. 2024;305:122432. 38176263 10.1016/j.biomaterials.2023.122432 PMC10994150

[3] Han X, Ye J, Huang R, Li Y, Liu J, Meng T, Song D. Pan-cancer analysis reveals interleukin-17 family members as biomarkers in the prediction for immune checkpoint inhibitor curative effect. Front Immunol. 2022;13:900273. 36159856 10.3389/fimmu.2022.900273 PMC9493092

[4] Bose R, Ma CX. Breast Cancer, HER2 Mutations, and Overcoming Drug Resistance. N Engl J Med. 2021;385(13):1241–3. 34551235 10.1056/NEJMcibr2110552

[5] Marques RF, Engler JB, Küchler K, Jones RA, Lingner T, Salinas G, Gillingwater TH, Friese MA, Duncan KE. Motor neuron translatome reveals deregulation of SYNGR4 and PLEKHB1 in mutant TDP-43 amyotrophic lateral sclerosis models. Hum Mol Genet. 2020;29(16):2647–61. 32686835 10.1093/hmg/ddaa140 PMC7530531

[6] Ma J, Wang H, Gui Z, Yang Y. Unveiling the role of SYNGR4 in breast cancer development: a novel target for immunotherapy. Front Oncol. 2024;14:1490073. 39902127 10.3389/fonc.2024.1490073 PMC11788336

[7] Yu B, Zhou Y, He J. TRIM13 inhibits cell proliferation and induces autophagy in lung adenocarcinoma by regulating KEAP1/NRF2 pathway. Cell Cycle. 2023;22(12):1496–513. 37245083 10.1080/15384101.2023.2216504 PMC10281484

[8] Gidado KI, Adeshakin FO, Rabiu L, Zhang Z, Zhang G, Wan X. Multifaceted roles of DLG3/SAP102 in neurophysiology, neurological disorders and tumorigenesis. Neuroscience. 2025;565:192–201. 39638232 10.1016/j.neuroscience.2024.11.081

[9] Li J, Xu Y, Zhu H, Wang Y, Li P, Wang D. The dark side of synaptic proteins in tumours. Br J Cancer. 2022;127(7):1184–92. 35624299 10.1038/s41416-022-01863-x PMC9519633

[10] Weinstein JN, Collisson EA, Mills GB, Shaw KR, Ozenberger BA, Ellrott K, Shmulevich I, Sander C, Stuart JM. The Cancer Genome Atlas Pan-Cancer analysis project. Nat Genet. 2013;45(10):1113–20. 24071849 10.1038/ng.2764 PMC3919969

[11] Consortium G. The Genotype-Tissue Expression (GTEx) project. Nat Genet. 2013;45(6):580–5. 23715323 10.1038/ng.2653 PMC4010069

[12] Forbes N. RNA-Based Stool Molecular Testing-A Novel Potential Option for Colorectal Cancer Screening. Gastroenterology. 2024;166(6):1190. 38147932 10.1053/j.gastro.2023.12.016

[13] Jenny K, Mayen B, Aditi V, David G. Clonal evolution and therapy resistance in the era of precision cancer medicine: evolutionary trajectories in pediatric cancer. Semin Cancer Biol. 2025;116(0):1–14. 10.1016/j.semcancer.2025.10.001 41067671

[14] Jia Xuan K, Jia Ning Nicolette Y, Ram Pravin KM, Xinyi W, Wei Heng C, Alvaro L-S, Kevin Kuang Wei T, Lih-Wen D, Dan G, Helene CB, et al. Colorectal Cancer at the Crossroads: The Good, the Bad, and the Future of Platinum-Based Drugs. Chem Rev. 2025;125:10248–341. 40985211 10.1021/acs.chemrev.5c00041 PMC12616619

[15] Jian Z, Zhaozhou R, Haibo W, Xing Y, Yang S, Jun L, Xiaoying L. Emerging Nanozyme Strategies for Precision Breast Cancer Treatment. Adv Sci (Weinh). 2025;12:e14085. 10.1002/advs.202514085 PMC12697830 41144769

[16] Qi W, Juan L, Ming Y, Jun Z, Yaxuan L, Jingjing Z, Hao J, Shuhua Y, Yinpeng L, Yuxin S, et al. Targeting AKR1B1 inhibits metabolic reprogramming to reverse systemic therapy resistance in hepatocellular carcinoma. Signal Transduct Target Ther. 2025;10(1):244. 40750772 10.1038/s41392-025-02321-9 PMC12317016

[17] Collaborators GCD. The global, regional, and national burden of cancer, 1990–2023, with forecasts to 2050: a systematic analysis for the Global Burden of Disease Study 2023. Lancet. 2025;406:10512. 10.1016/S0140-6736(25)01635-6 PMC12687902 41015051

[18] Liu C, Xianyu B, Dai Y, Pan S, Li T, Xu H. Intracellular Hyperbranched Polymerization for Circumventing Cancer Drug Resistance. ACS Nano. 2023;17(12):11905–13. 37285408 10.1021/acsnano.3c03512

[19] Amanda MM, Kelsey LS, Michael JM. Engineering Nanoparticles for Gynecologic Cancer Therapy. ACS Nano. 2025;19(34):30758–85. 40829053 10.1021/acsnano.5c07748

[20] Mingyang J, Jinlong W, Yize L, Ke Z, Tao W, Zhandong B, Shenyi L, Raquel Alarcón R, Ruqiong W, Mingtao Z, et al. EMT and cancer stem cells: Drivers of therapy resistance and promising therapeutic targets. Drug Resist Updat. 2025;83:10276. 10.1016/j.drup.2025.101276 PMC13560432 40743619

[21] Yiqun Z, Fengju C, Darshan SC, Sooryanarayana V, Chad JC. Proteogenomic characterization of 2002 human cancers reveals pan-cancer molecular subtypes and associated pathways. Nat Commun. 2022;13(1):2669. 35562349 10.1038/s41467-022-30342-3 PMC9106650

[22] Francisco S-V, Marco M, Joshua A, Walid KC, Augustin L, Konnor CL, Sofia D, David LL, Havish SK, Sadegh S, et al. Oncogenic Signal Pathways Cancer Genome Atlas Cell. 2018;173(2):321–37. 29625050 10.1016/j.cell.2018.03.035 PMC6070353

[23] Syed H, Alan M, Ruud GPM, vS LMW, Simon W, Adrian LH. Francesca M B: Genomic alterations underlie a pan-cancer metabolic shift associated with tumour hypoxia. Genome Biol. 2016;17:1. 27358048 10.1186/s13059-016-0999-8 PMC4926297

[24] Kathleen W, Bianca D, Krzysztof JS, Laura L, Predrag J, Shan C, Ranveer P, Julia AV, Tyler TC, David P, et al. Epigenetic alterations facilitate transcriptional and translational programs in hypoxia. Nat Cell Biol. 2025;16:1–7. 10.1038/s41556-025-01786-8 PMC12611764 41102449

[25] Naomi MP, Zachary S, Quanming S, Julia AB, Dmytro D, Katerina K, Kevin RP, Brian RS, Alexander M, Howard YC. Bidirectional epigenetic editing reveals hierarchies in gene regulation. Nat Biotechnol. 2024;43(3):355–68. 38760566 10.1038/s41587-024-02213-3 PMC11569274

[26] Kai L, Lulu W, Ranran W, Li L, Shiyu S, Fei W, Meiwei H, Wenyuan P, Jinglin W, Junaid W, et al. Disrupted methionine cycle triggers muscle atrophy in cancer cachexia through epigenetic regulation of REDD1. Cell Metab. 2024;37(2):460–76. 39729999 10.1016/j.cmet.2024.10.017

[27] Nguyen TBN, Sira G, Rutger NUK, Lotte MB, Hannah N, Ninouk A, Max AB, Marlies CL, Can G. Edwin C A S: Lactate controls cancer stemness and plasticity through epigenetic regulation. Cell Metab. 2025;37(4):903–19. 39933514 10.1016/j.cmet.2025.01.002

[28] Han X, Song D. Using a Machine Learning Approach to Identify Key Biomarkers for Renal Clear Cell Carcinoma. Int J Gen Med. 2022;15:3541–58. 35392028 10.2147/IJGM.S351168 PMC8980298

[29] Wang Y, Lu K, Xu Y, Xu S, Chu H, Fang X. Antibody-drug conjugates as immuno-oncology agents in colorectal cancer: targets, payloads, and therapeutic synergies. Front Immunol. 2025;16:1678907. 41256852 10.3389/fimmu.2025.1678907 PMC12620403

[30] Wang P, Xu S, Guo Q, Zhao Y. Discovery of PAK2 as a Key Regulator of Cancer Stem Cell in Head and Neck Squamous Cell Carcinoma Using Multi-Omic Techniques. Stem Cells Int. 2025;2025:1325262. 41311809 10.1155/sci/1325262 PMC12657082

[31] Xu S, Guo Q, Lu Z. Physical Activity, Lifestyle, and Cancer Risk. JAMA Oncol. 2026;12(4):425–6. 41746622 10.1001/jamaoncol.2025.6508

[32] Michael AH, Peter S, Balaji V, Megan MROM, Jasmine K, Scott NM, Laura KM, Roberto S, Sherene L. Towards targeting the breast cancer immune microenvironment. Nat Rev Cancer. 2024;24(8):554–77. 38969810 10.1038/s41568-024-00714-6

[33] Jianxia L, Cheng W, Huabin H, Ge Q, Xueqian W, Fan B, Jianwei Z, Yue C, Yan H, Chao W, et al. Remodeling of the immune and stromal cell compartment by PD-1 blockade in mismatch repair-deficient colorectal cancer. Cancer Cell. 2023;41(6):1152–69. 37172580 10.1016/j.ccell.2023.04.011

[34] Tri Giang P, Peter IC. The dormant cancer cell life cycle. Nat Rev Cancer. 2020;20(7):398–411. 32488200 10.1038/s41568-020-0263-0

[35] Jing L, Yunhua P, Wenyi W. Cell cycle on the crossroad of tumorigenesis and cancer therapy. Trends Cell Biol. 2021;32(1):30–44. 34304958 10.1016/j.tcb.2021.07.001 PMC8688170

[36] Agnieszka KW, Vishnu K, Ioannis S, Erik SK. Cancer cell cycle dystopia: heterogeneity, plasticity, and therapy. Trends Cancer. 2022;8(9):711–25. 35599231 10.1016/j.trecan.2022.04.006 PMC9388619

[37] Solène H, Andrea S, Gabriele BM, Marine G, Kotryna V, Marie D, Annapaola A, Adib K, Gianluca P, Mathieu D, et al. Chromosome mis-segregation triggers cell cycle arrest through a mechanosensitive nuclear envelope checkpoint. Nat Cell Biol. 2025;27(1):73–86. 39779939 10.1038/s41556-024-01565-x PMC11735390

[38] Jingyi W, Liu L, Xinyu G, Xiyu L, Yitian D, Zijun M, Shengzhe H, Junjian L, Dongliang W, Yu Q, et al. A novel pathway for stemness propagation and chemoresistance in non-small cell lung cancer via phosphorylated PKM2-loaded small extracellular vesicles. Theranostics. 2025;15(8):3439. 40093893 10.7150/thno.103722 PMC11905138

[39] Steele EJ. Somatic hypermutation in immunity and cancer: Critical analysis of strand-biased and codon-context mutation signatures. DNA Repair (Amst). 2016;45:1–24. 27449479 10.1016/j.dnarep.2016.07.001

[40] Sivanandam A, Murthy S, Kim SH, Barrack ER, Veer Reddy GP. Role of androgen receptor in prostate cancer cell cycle regulation: interaction with cell cycle regulatory proteins and enzymes of DNA synthesis. Curr Protein Pept Sci. 2010;11(6):451–8. 20491624 10.2174/138920310791824075

[41] Shilkin ES, Boldinova EO, Stolyarenko AD, Goncharova RI, Chuprov-Netochin RN, Smal MP, Makarova AV. Translesion DNA Synthesis and Reinitiation of DNA Synthesis in Chemotherapy Resistance. Biochem (Mosc). 2020;85(8):869–82. 10.1134/S0006297920080039 33045948

[42] Yang Y, Badura ML, O’Leary PC, Delavan HM, Robinson TM, Egusa EA, Zhong X, Swinderman JT, Li H, Zhang M, et al. Transcription and DNA replication collisions lead to large tandem duplications and expose targetable therapeutic vulnerabilities in cancer. Nat Cancer. 2024;5(12):1885–901. 39558146 10.1038/s43018-024-00848-4 PMC11671220

[43] Shen JZ, Qiu Z, Wu Q, Finlay D, Garcia G, Sun D, Rantala J, Barshop W, Hope JL, Gimple RC, et al. FBXO44 promotes DNA replication-coupled repetitive element silencing in cancer cells. Cell. 2021;184(2):352–e369323. 33357448 10.1016/j.cell.2020.11.042 PMC8043252

